# The Effect of Socioeconomic Status on All-Cause Maternal Mortality: A Nationwide Population-Based Cohort Study

**DOI:** 10.3390/ijerph17124606

**Published:** 2020-06-26

**Authors:** Wonjeong Jeong, Sung-In Jang, Eun-Cheol Park, Jin Young Nam

**Affiliations:** 1 Department of Public Health, Graduate School, Yonsei University, Seoul 03722, Korea; wjjeong@yuhs.ac; 2 Institute of Health Services Research, Yonsei University, Seoul 03722, Korea; JANGSI@yuhs.ac (S.-I.J.); ecpark@yuhs.ac (E.-C.P.); 3 Department of Preventive Medicine, College of Medicine, Yonsei University, Seoul 03722, Korea; 4 Department of Public Health Science, BK21PLUS Program in Embodiment, Health-Society Interaction, Graduate School, Korea University, Seoul 02841, Korea

**Keywords:** maternal death, maternal mortality, socioeconomic status, primipara, childbirth

## Abstract

Improving maternal health is one of the 13 targets of Sustainable Development Goal 3; consequently, preventing maternal death, which usually occurs in women’s prime productive years, is an important issue that needs to be addressed immediately. This study examines the association between socioeconomic status and all-cause maternal mortality in South Korea and provides evidence of preventable risk factors for maternal death. For this population-based retrospective cohort study, data on 3,334,663 nulliparous women were extracted from the Korean National Health Insurance Service database between 2003 and 2018. The outcome variables were all-cause maternal mortality within six weeks and a year after childbirth. A log-binomial regression model determined the association between maternal mortality and income-level adjusted covariates. Women with lower income levels had higher risk of maternal death within six weeks (risk ratio (RR) = 2.42, 95% confidence interval (CI) = 1.65–3.53) and within one year (RR = 1.83, 95% CI = 1.47–2.28), especially those who were aged 35–39 years, lived in rural areas, delivered via cesarean section, and had maternal comorbidities. The study identifies a significant relationship between South Korean primiparas’ socioeconomic status and maternal death within six weeks or one year after childbirth, suggesting interventions to alleviate the risk of maternal death.

## 1. Introduction

The World Health Organization (WHO) announced that to improve maternal health, barriers that limit access to quality maternal health services must be identified and addressed at all levels of the health system [1]. Improving maternal health is one of the 13 targets for Sustainable Development Goal (SDG) 3 and an important issue that needs to be addressed immediately [2]. The first target of the SDG 3 is to reduce the global maternal mortality ratio to less than 70 per 100,000 live births by 2030 [2,3]. Although the maternal mortality ratio dropped by about 38% worldwide between 2000 and 2017, approximately 800 women died every day in 2017 from preventable causes related to pregnancy such as severe bleeding, infections, complications from delivery, and pre-eclampsia [1,4]. To avoid these preventable causes of maternal death, skilled health professionals and improved quality care during and after childbirth are needed [1,5]. The estimated lifetime risk of maternal mortality is substantially higher in low-income countries [3].

A pregnancy-related death can occur during pregnancy, at delivery, and even up to a year afterward [6]. Maternal death, however, has been defined as the death of a woman while pregnant or within 42 days of the termination of pregnancy [3]. Because one in three pregnancy-related deaths occur one week to one year after delivery, it is critically important to pay attention to maternal deaths that occurs within not only six weeks, but also one year after delivery [6]. Maternal deaths are caused not only by the risks attributable to pregnancy and childbirth, but also the poor-quality care from health services, which may be impacted by income level and residential areas [7]. However, due to complexities in the definitions of maternal death and the relationship between different conditions that may be reported as causes of death, more accurate identification and classification of the actual cause is needed [8].

Socioeconomic status is one risk factor for maternal mortality. Some low-income countries (LICs) have more than 100 times the maternal mortality ratio than high income countries (HICs) caused by pre-eclampsia/eclampsia [9], anesthesia-related problems, obstetric complications, or caesarean section difficulties (C-sections) [10]. Moreover, residential areas such as rural areas influence adverse maternal health outcome due to disparity of maternal health care utilization, such as prenatal care at a health facility or facility-based delivery [11]. Maternal mortality in HICs has been dramatically reduced in the last few decades but it still occurs at around 10 per 100,000 birth in HICs [9]. Numerous studies have explored the risk factors for maternal mortality in LICs, but few studies have been conducted on the association between socioeconomic status and maternal mortality in HICs.

South Korea had the sixth highest maternal mortality ratio in 2017 among Organization for Economic Co-operation and Development (OECD) countries [12], even though the country has high accessibility to obstetricians and obstetric facilities. To reduce preventable pregnancy-related deaths [6,7], factors that could lead to maternal mortality need to be found, including maternal clinical status and socioeconomic status as they relate to the healthcare system. This could prevent maternal death by providing information about key environmental risk factors. Consequently, the current study examines the association between socioeconomic status and all-cause maternal mortality among nulliparous women in South Korea.

## 2. Methods

### 2.1. Data and Study Participants

This study used nationwide retrospective claim data from the Korean National Health Insurance Service (NHIS). This database comprised information on healthcare utilization of the entire population of South Korean, including age, sex, residential area, type of health insurance, income, medical diagnosis and procedure codes, prescription drugs, individual total medical costs, and hospital information. The NHIS can follow up the patient, record clinical and provision’s characteristics over time, present the epidemiologic causes of disease, and inform the development of healthcare policies. This database includes unique de-identified numbers for the patients’ in order to mask personally identifiably information [13]. The mortality dates of individuals in this cohort were provided by the Korean National Statistical Office.

This study focused on women aged between 15 and 50, who were nulliparous, and who were enrolled in the Korean National Health Insurance Service database for at least 280 days before childbirth until six weeks after childbirth between January 1, 2003 and November 19, 2018. Childbirth was defined based on hospitalization delivery records with delivery-related diagnosis or procedure codes for spontaneous vaginal, instrumental or C-sections deliveries. Therefore, among 3,531,805 nulliparous women, we excluded women who delivered in 2002 and after November 20, 2018, as well as those who did not have records for delivery admission (*n* = 197,297). The total number of childbirth cases in this cohort was 3,334,663.

### 2.2. Variables

#### 2.2.1. Maternal Mortality

Two outcome variables were examined: (1) maternal mortality within 42 days (6 weeks) after childbirth date and (2) maternal mortality within a year after childbirth date. Maternal mortality included all-cause death because the database did not include cause of death.

#### 2.2.2. Income Level

The variable of interest was income level. The NHIS database includes the beneficiaries’ premium as a proxy for the income of subscribers or their families. The NHIS premiums are mandatory and imposed based on monthly salary, taxable income, and assets; therefore, it was considered as the level of individuals’ income. In this study, individuals’ income levels were categorized as follow: (1) low income (below 25th percentile), (2) mid-low income (25th–50th percentile), (3) mid-high income (51st–75th percentile), and (4) high income (76th–100th percentile).

#### 2.2.3. Covariates

General characteristics of the study population included maternal age (15–49 years), type of insurance (self-employed, employee insured, or medical aid), residential area (Seoul metropolitans, other metropolitans, small cities, or rural areas), and working status (working or not working). Maternal obstetric characteristics included mode of delivery (spontaneous vaginal, instrumental, or C-sections delivery), preterm birth status (no or yes), and multiple birth status (no or yes). Prenatal care was estimated using the Kessner Adequacy of Prenatal Care Index (adequate, intermediate or inadequate) [14], and maternal comorbidities during pregnancy were estimated using Howell’s study (0 or +1) [15]. Hospital characteristics included the type of hospital—categorized by the number of beds (more than 500, 100–499, 30–99, or less than 30 beds)—and year of delivery, which was used as an adjustment factor.

### 2.3. Statistical Analysis

The study estimated the distribution of the general characteristics of the study population who delivered between 2003 and 2018. The association between income level and maternal mortality within six weeks and one year after childbirth were estimated adjusted risk ratio (RR) and 95% confidence intervals (CIs) using a log-binomial regression model adjusted all covariates. The log-binomial regression model was generated for stratified analysis by maternal age, residential area, mode of delivery, and maternal comorbidities and tested for interactions. All statistical analyses were conducted using SAS 9.4 (SAS Institute, Inc., Cary, NC, USA).

This study adhered to the tenets of the Declaration of Helsinki, and the study design was reviewed and approved by the Korea University, Institutional Review Board (KUIRB-2020-0097-01).

## 3. Results

Table 1 presents the general characteristic of the study population according to household income level. In total, 3,334,663 nulliparous women were included, 353 (0.01%) and 933 (0.03%) died within six weeks and within one year after delivery, respectively, 79.4% of these women delivered at the age of 25–34 years, 47.6% of women lived in metropolitans, 38.5% had Caesarean-section delivery, and 38.1% experienced maternal comorbidities. 

Table 2 shows the association between maternal death and the risk factors adjusted for all covariates. Individuals of lower income levels had higher risk of maternal death within six weeks (lowest income: RR = 2.42, 95% CI = 1.65–3.53), and within one year (lowest income: RR = 1.83, 95% CI = 1.47–2.28). Women who lived in small cities and rural areas had a higher risk of maternal death within one year after childbirth (small cities: RR = 1.23, 95% CI = 1.04–1.46; rural areas: RR = 1.37, 95% CI = 1.05–1.79). Moreover, higher risk of maternal death in both periods was found in women who had C-sections (maternal death within six weeks: RR = 3.10, 95% CI = 2.26–4.42; maternal death within one year: RR = 2.02, 95% CI = 1.70–2.41) and those who had maternal comorbidities (within six weeks: RR = 6.05, 95% CI = 4.68–7.83; within one year: RR = 2.41, 95% CI = 2.09–2.77).

The characteristics were adjusted for maternal age, type of insurance, residential areas, working status, mode of delivery, preterm birth, prenatal care, multiple birth, maternal comorbidities, type of hospital, and year of delivery.

Table 3 shows the association between income level and maternal mortality stratified by maternal age, residential areas, mode of delivery, and maternal comorbidities. We found a stronger association between low income level and risk of maternal mortality within six weeks after childbirth for those aged 35–39 years (RR = 3.40, 95% CI = 1.59–7.29), all residential areas (except the lowest income level in rural areas), C-section delivery (RR = 3.23, 95% CI = 1.97–5.27), and having maternal comorbidities (RR = 2.87, 95% CI = 1.81–4.55). However, the interactions were statistical significant only for maternal age in maternal mortality within 6 weeks after childbirth (*p*-interaction = 0.04). Moreover, there were statistically significant associations between low income levels and maternal mortality within one year after childbirth in women aged 25–39, living in metropolitan areas and small cities, having had a C-section, and having comorbidities. The effects of income level on maternal mortality within 1 year were different depending on residential area (*p*-interaction = 0.02), and having maternal comorbidities (*p*-interaction = 0.03).

## 4. Discussion

As maternal health is an important issue that requires immediate attention, eliminating preventable maternal death is a primary goal of maternal death surveillance and response [16]. It is important to understand socioeconomic factors to not only reduce maternal death but also design effective health and hospital policies [17]. Therefore, the purpose of this study is to reveal the association between socioeconomic status and all-cause maternal mortality, thereby providing evidence to protect women who are at high risk of maternal death. Our finding suggests that socioeconomic status—especially income level—is highly associated with all-cause maternal mortality.

It is well known that low-income individuals are at greater risk for maternal death than is the higher-income segment of the population [18,19]. Income is a key risk factor for maternal death, as it is highly associated with three delays (decision to seek care, access to care, and timeliness and quality of care) [20,21,22]. The reason for the delay in decision to seek care is fear of the hospital cost or lack of an available decision maker [22]. Moreover, difficulty in transport could delay arrival at the appropriate facility [22]. As low-income women usually live in rural areas, this could be the main factor for the difficulty with transport [23]. In such areas, women who need urgent care may not have sufficient time to reach the facility. Therefore, delays due to care access could more strongly affect maternal death for low-income individuals. Similarly, delays in receiving adequate care in the facility is usually due to difficulty getting equipment or blood supplies, which could affect maternal death in low-income individuals [22]. Hospitals in metropolitan areas have more new equipment that can easily provide adequate care. Difficulty accessing such equipment could be related to severe bleeding, a preventable cause of maternal death [5]. Furthermore, risk of maternal death also increases when the economy worsens [24]. For low-income women, the increased likelihood of experiencing both catastrophic costs and diminished productivity has meant that maternal death is higher in low-income households [24,25]. Our study also found that compared to the high-income group, the low-income group had a higher risk of maternal death within six weeks and within one year after childbirth. Additionally, we found that those who were not working had higher risk of maternal death both within six weeks and within one year after childbirth. As working is highly associated with increased income, it should decrease the risk of maternal death due to increased income.

Because maternal death is highly associated with women’s access to medical care for complications, residential areas could be associated with higher risk of maternal death [23,26]. Our study reveals that women who live in rural areas have a higher risk of maternal mortality compared to those who live in the capital city. However, in the stratified analysis, there was no statistically significant association among those living in rural areas. This result suggests that there is a disparity between urban and rural areas that might not exist among rural areas based on income levels because women who live in rural areas face similar circumstances regarding accessibility of obstetric facilities regardless of income. Disparities between South Korea’s urban and rural populations have been reported with regard to health status and access to healthcare and resources [27,28]. Additionally, due to the high mean age of mothers at childbirth, the risk of maternal death is further increased for those who live in metropolitans in South Korea [12,29,30].

Maternal death and morbidity is approximately five times greater with C-sections than with vaginal deliveries, because there is a higher risk of hemorrhage and sepsis with regard to the former [31,32]. Our study shows that those who underwent C-sections for their first childbirth had a higher risk of maternal death within six weeks and within one year. Moreover, those who had maternal comorbidities also had a higher risk of maternal death. Although C-sections play an important role as lifesaving interventions, they are a type of major abdominal surgery accompanied by more potential complications than accompany a vaginal delivery [33]. Due to C-sections later on, the risk of maternal death and severe maternal morbidity increases in subsequent pregnancies cumulatively [31]. Therefore, because C-sections could be associated with adverse maternal health outcome, they should only be provided when necessary [26]. Interestingly, there was a positive association between low income level and high risk of maternal mortality among women with C-sections and those having maternal comorbidities. Although we cannot explain the exact etiology of these results, women with low income levels might experience an underuse of maternal health care, such as prenatal or postnatal care, due to financial burdens [11]. Women who had C-sections or comorbidities are likely to be categorized as having high-risk pregnancies, and increasing their cost (especially out-of-pocket expenditures) is unavoidable, even though South Korea has national health care. Thus, paradoxically, low-income women with high-risk pregnancies may face lower maternal mortality risk, as they have no choice but to receive more frequent care. To improve maternal health outcomes during/after childbirth, financial support for maternal care is needed among low-income pregnant women.

It should be noted that the current study has several limitations. First, as it includes all-cause maternal mortality, it could not confirm whether the mothers died as a result of direct obstetric deaths. However, because the definition of maternal death is the death of a woman while pregnant or within 42 days of termination of pregnancy, considering death within six weeks after childbirth can be interpreted as an obstetric death [34]. Second, because there were not many cases, there were very few participants who succumbed to maternal mortality within six weeks or one year after delivery. However, as this study was based on the NHIS data of all of South Korean primiparas, this could be representative of the South Korean population. Lastly, this study only included primiparas. Therefore, further studies should include women who experience more than one childbirth.

The strengths of our study are as follows. It used national data, which most closely represents all South Korean women who experience pregnancy. Therefore, the findings could inform the formulation of policies to protect pregnant women who are in danger. Moreover, as the South Korean maternal age at childbirth has increased, so has the risk of maternal death [35]. Therefore, providing information about South Korean maternal death using recent Korean NHIS claim data is important. Lastly, analyzing with the data about both maternal deaths within 6 weeks and within 1 year could provide more specific information of maternal death.

## 5. Conclusions

The current study identified a significant relationship between socioeconomic status and maternal death within six weeks and within one year among South Korean primiparas. This finding suggests that people who had lower income levels, lived in rural areas, delivered via cesarean section, and had maternal comorbidities had a higher risk of maternal death. Moreover, among women who lived in large or small cities, those who had C-sections or had maternal comorbidities and had a low income level had a higher risk of maternal death than those with a high income level. This shows that more attention should be paid to decrease the risk of maternal death based on individuals’ low socioeconomic factors. Considering that maternal deaths usually occur in women’s prime productive years, this is an urgent care need [25]. Therefore, our study suggests that policy makers should encourage governments and local health authorities to allocate budgets for the improvement of quality of childbirth care in vulnerable group to alleviate the risk of maternal death.

## Figures and Tables

**Table 1:** General characteristic of study population.

	Household Income								Total	
	Q1		Q2		Q3		Q4			
	*N*	(%)	*N*	(%)	*N*	(%)	*N*	(%)	*N*	(%)
Maternal mortality within 6 weeks										
No	712,181	(21.4)	934,801	(28.0)	1,141,944	(34.3)	545,384	(16.4)	3,334,310	(100.0)
Yes	103	(29.2)	101	(28.6)	109	(30.9)	40	(11.3)	353	(0.0)
Maternal mortality within 1 year										
No	712,013	(21.4)	934,643	(28.0)	1,141,781	(34.3)	545,293	(16.4)	3,333,730	(100.0)
Yes	271	(29.1)	259	(27.8)	272	(29.2)	131	(14.0)	933	(0.0)
Age										
15–19	8547	(38.6)	6066	(27.4)	4863	(21.9)	2685	(12.1)	22,161	(0.7)
20–24	82,388	(33.5)	75,140	(30.6)	57,490	(23.4)	30,750	(12.5)	245,768	(7.4)
25–29	297,788	(24.5)	392,193	(32.2)	379,210	(31.2)	147,843	(12.2)	1,217,034	(36.5)
30–34	247,843	(17.3)	371,285	(26.0)	554,448	(38.8)	256,196	(17.9)	1,429,772	(42.9)
35–39	66,037	(17.8)	79,919	(21.6)	131,580	(35.5)	93,213	(25.1)	370,749	(11.1)
40–44	9358	(19.7)	9938	(20.9)	14,017	(29.5)	14,220	(29.9)	47,533	(1.4)
45+	323	(19.6)	361	(21.9)	445	(27.0)	517	(31.4)	1646	(0.1)
Type of Insurance										
Self-employed	192,498	(22.7)	238,148	(28.1)	246,191	(29.1)	170,341	(20.1)	847,178	(25.4)
Employees	506,078	(20.5)	696,754	(28.2)	895,862	(36.2)	375,083	(15.2)	2,473,777	(74.2)
Medical aids	13,708	(100.0)	0	(0.0)	0	(0.0)	0	(0.0)	13,708	(0.4)
Residential areas										
Seoul metropolitan area	125,958	(16.4)	205,117	(36.3)	278,116	(36.3)	157,821	(20.6)	767,012	(23.0)
Other metropolitan areas	185,561	(22.6)	241,853	(33.9)	277,992	(33.9)	115,560	(14.1)	820,966	(24.6)
Small cities	339,180	(22.0)	430,280	(27.9)	527,291	(34.2)	247,153	(16.0)	1,543,904	(46.3)
Rural areas	61,585	(30.4)	57,652	(28.4)	58,654	(28.9)	24,890	(12.3)	202,781	(6.1)
Working status										
Working	388,195	(25.5)	484,546	(31.8)	504,894	(33.1)	146,527	(9.6)	1,524,162	(45.7)
Not working	324,089	(17.9)	450,356	(24.9)	637,159	(35.2)	398,897	(22.0)	1,810,501	(54.3)
Mode of delivery										
Spontaneous vaginal delivery	212,734	(21.1)	283,464	(28.1)	347,942	(34.5)	165,745	(16.4)	1,009,885	(30.3)
Instrumental delivery	225,129	(21.6)	292,984	(28.1)	357,996	(34.4)	166,181	(15.9)	1,042,290	(31.3)
Cesarean section	274,421	(21.4)	358,454	(28.0)	436,115	(34.0)	213,498	(16.7)	1,282,488	(38.5)
Preterm birth										
No	696,139	(21.4)	914,927	(28.1)	1,116,091	(34.3)	531,301	(16.3)	3,258,458	(97.7)
Yes	16,145	(21.2)	19,975	(26.2)	25,962	(34.1)	14,123	(18.5)	76,205	(2.3)
Prenatal care										
Adequacy	603,111	(20.8)	808,661	(28.1)	1,009,233	(34.8)	482,523	(16.6)	2,903,528	(87.1)
Intermediate	94,884	(24.8)	112,202	(29.3)	119,376	(31.2)	56,568	(14.8)	383,030	(11.5)
Inadequacy	142,89	(29.7)	14,039	(29.2)	13,444	(28.0)	6333	(13.2)	48,105	(1.4)
Multiple birth										
No	700,305	(21.4)	919,814	(28.2)	1,116,645	(34.2)	528,913	(16.2)	3,265,677	(97.9)
Yes	11,979	(17.4)	15,088	(21.9)	25,408	(36.8)	16,511	(23.9)	68,986	(2.1)
Maternal comorbidities										
No	459,537	(22.3)	595,813	(28.9)	691,905	(33.5)	315,584	(15.3)	2,062,839	(61.9)
Yes	252,747	(19.9)	339,089	(26.7)	450,148	(35.4)	229,840	(18.1)	1,271,824	(38.1)
Type of hospital										
More than 500 beds	36,833	(17.7)	46,270	(22.2)	76,453	(36.6)	49,138	(23.6)	208,694	(6.3)
100–499 beds	72,230	(19.2)	95,109	(25.3)	134,393	(35.7)	74,561	(19.8)	376,293	(11.3)
30–99 beds	277,987	(20.5)	382,313	(28.2)	474,664	(35.1)	218,689	(16.2)	13,536,53	(40.6)
Less than 30 beds	325,234	(23.3)	411,210	(29.5)	456,543	(32.7)	203,036	(14.5)	1,396,023	(41.9)
Total	712,284	(21.4)	934,902	(28.0)	1,142,053	(34.3)	545,424	(16.4)	3,334,663	(100.0)

**Table 2:** The association between maternal mortality and maternal characteristics adjusted for all covariates.

	Total	Maternal Mortality within 6 Weeks (2003–2018)			Maternal Mortality within 1 Year (2003–2018)		
		# of cases	RR	(95% CI)	# of cases	RR	(95% CI)
Household Income							
1Q (low)	712,284	103	2.42	(1.65, 3.53)	271	1.83	(1.47, 2.28)
2Q	934,902	101	1.88	(1.30, 2.73)	259	1.41	(1.14, 1.75)
3Q	1,142,053	109	1.57	(1.09, 2.26)	272	1.16	(0.94, 1.44)
4Q (high) (reference)	545,424	40	1.00		131	1.00	
Age							
15–19	22,161	3	1.70	(0.48, 6.03)	8	0.88	(0.42, 1.86)
20–24 (reference)	245,768	15	1.00		71	1.00	
25–29	1,217,034	108	1.65	(0.95, 2.85)	291	0.98	(0.75, 1.27)
30–34	1,429,772	135	1.65	(0.96, 2.85)	379	1.08	(0.83, 1.40)
35–39	370,749	80	2.94	(1.67, 5.19)	150	1.36	(1.01, 1.82)
40+	49,179	12	2.33	(1.08, 5.05)	34	1.77	(1.16, 2.69)
Type of Insurance							
Self-employed	847,178	126	1.29	(1.03, 1.63)	341	1.44	(1.25, 1.66)
Employees (reference)	2,473,777	221	1.00		575	1.00	
Medical aids	13,708	6	3.18	(1.35, 7.53)	17	3.36	(2.01, 5.61)
Residential areas							
Seoul metropolitan(reference)	767,012	81	1.00		199	1.00	
Other metropolitans	820,966	78	1.03	(0.75, 1.41)	207	1.06	(0.87, 1.29)
Small cities	1,543,904	166	1.16	(0.88, 1.52)	451	1.23	(1.04, 1.46)
Rural areas	202,781	28	1.28	(0.83, 1.98)	76	1.37	(1.05, 1.79)
Working status							
Working (reference)	1,524,162	129	1.00		340	1.00	
Not working	1,810,501	224	1.39	(1.11, 1.75)	593	1.37	(1.19, 1.58)
Mode of delivery							
Spontaneous vaginal delivery (reference)	1,009,885	49	1.00		178	1.00	
Instrumental delivery	1,042,290	58	1.12	(0.77, 1.65)	220	1.18	(0.97, 1.44)
Cesarean section	1,282,488	246	3.10	(2.26, 4.42)	535	2.02	(1.70, 2.41)
Preterm birth							
No (reference)	3,258,458	333	1.00		884	1.00	
Yes	76,205	20	1.24	(0.76, 2.00)	49	1.28	(0.94, 1.74)
Prenatal care							
Adequacy (reference)	2,903,528	280	1.00		766	1.00	
Intermediate	383,030	67	1.53	(1.13, 2.07)	148	1.21	(1.00, 1.47)
Inadequacy	48,105	6	1.37	(0.59, 3.15)	19	1.31	(0.82, 2.10)
Multiple birth							
No (reference)	3,265,677	336	1.00		899	1.00	
Yes	68,986	17	0.91	(0.55, 1.51)	34	0.84	(0.59, 1.20)
Maternal comorbidities							
No (reference)	2,062,839	87	1.00		397	1.00	
Yes	1,271,824	266	6.05	(4.68, 7.83)	536	2.41	(2.09, 2.77)
Type of hospital							
More than 500 beds	208,694	68	3.29	(2.32, 4.67)	160	3.01	(2.49, 3.72)
100–499 beds	376,293	71	2.17	(1.55, 3.05)	165	1.83	(1.49, 2.24)
30–99 beds (reference)	1,353,653	77	1.00		260	1.00	
Less than 30 beds	1,396,023	137	1.69	(1.27, 2.25)	348	1.23	(1.04, 1.45)

RR: risk ratio; # of cases: number of maternal mortality.

**Table 3:** The associations between maternal mortality and income levels, stratified by maternal age, residential areas, mode of delivery, and maternal comorbidities after adjusting for all covariates.

	Household Income								
	1Q (Lowest)		2Q		3Q		4Q (Highest) (Reference)		*p* for Interaction
	RR	(95% CI)	RR	(95% CI)	RR	(95% CI)	RR	(95% CI)	
Maternal mortality within 6 weeks after childbirth									
Age *									0.0395
15–19	-		-		-		1.00		
20–24	-		-		-		1.00		
25–29	2.79	(1.34, 5.80)	1.66	(0.79, 3.51)	1.07	(0.49, 2.34)	1.00		
30–34	1.29	(0.71, 2.35)	1.29	(0.74, 2.23)	1.35	(0.81, 2.24)	1.00		
35–39	3.40	(1.59, 7.29)	2.81	(1.33, 5.93)	1.58	(0.74, 3.37)	1.00		
40+	-		-		-		1.00		
Residential areas **									0.9392
Seoul metropolitan	2.48	(1.19, 5.17)	1.41	(0.67, 2.98)	1.78	(0.91, 3.49)	1.00		
Other metropolitans	2.35	(1.03, 5.37)	1.76	(0.78, 4.00)	1.42	(0.63, 3.21)	1.00		
Small cities	2.48	(1.42, 4.33)	2.10	(2.10, 3.62)	1.54	(0.90, 2.65)	1.00		
Rural areas	2.24	(0.47, 10.78)	2.56	(2.56, 11.95)	1.46	(0.29, 7.35)	1.00		
Mode of delivery ^†^									0.1939
Spontaneous vaginal delivery	1.92	(0.70, 5.25)	1.45	(0.54, 3.94)	1.79	(0.71, 4.53)	1.00		
Instrumental delivery	1.13	(0.52, 2.47)	0.78	(0.35, 1.71)	0.65	(0.30, 1.40)	1.00		
Cesarean section	3.23	(1.97, 5.27)	2.60	(1.61, 4.22)	2.01	(1.24, 3.24)	1.00		
Maternal comorbidities ^‡^									0.1789
No	1.60	(0.81, 3.14)	1.10	(0.56, 2.16)	0.99	(0.51, 1.92)	1.00		
Yes	2.87	(1.81, 4.55)	2.32	(1.48, 3.65)	1.88	(1.21, 2.93)	1.00		
Maternal mortality within 1 year after childbirth									
Age *									0.1173
15–19	-		-		-		1.00		
20–24	1.86	(0.74, 4.70)	1.64	(0.66, 4.09)	1.31	(0.50, 3.43)	1.00		
25–29	2.07	(1.36, 3.14)	1.42	(0.93, 2.16)	1.07	(0.70, 1.65)	1.00		
30–34	1.46	(1.05, 2.04)	1.09	(0.79, 1.50)	1.10	(0.82, 1.49)	1.00		
35–39	2.15	(1.27, 3.63)	2.23	(1.35, 3.67)	1.13	(0.68, 1.89)	1.00		
40+	2.56	(0.84, 7.77)	2.33	(0.78, 6.97)	1.75	(0.58, 5.23)	1.00		
Residential areas **									0.0177
Seoul metropolitan	1.78	(1.14, 2.77)	1.07	(0.68, 1.66)	1.27	(0.85, 1.90)	1.00		
Other metropolitans	2.90	(1.73, 4.88)	1.90	(1.12, 3.20)	1.26	(0.74, 2.14)	1.00		
Small cities	1.60	(1.18, 2.19)	1.29	(0.95, 1.75)	1.10	(0.82, 1.48)	1.00		
Rural areas	1.24	(0.53, 2.89)	2.03	(0.92, 4.48)	1.03	(0.44, 2.41)	1.00		
Mode of delivery ^†^									0.3537
Spontaneous vaginal delivery	1.85	(1.10, 3.12)	1.50	(0.90, 2.51)	1.48	(0.90, 2.43)	1.00		
Instrumental delivery	1.34	(0.87, 2.06)	1.24	(0.82, 1.88)	0.80	(0.53, 1.22)	1.00		
Cesarean section	2.08	(1.56, 2.78)	1.47	(1.10, 1.96)	1.26	(0.95, 1.67)	1.00		
Maternal comorbidities ^‡^									0.0267
No	1.49	(1.10, 2.05)	0.98	(0.72, 1.35)	0.88	(0.65, 1.21)	1.00		
Yes	2.13	(1.58, 2.88)	1.86	(1.39, 2.49)	1.43	(1.08, 1.91)	1.00		

RR: risk ratio. * Adjusted for type of insurance, residential area, working status, mode of delivery, preterm birth, prenatal care, multiple birth, maternal comorbidities, type of hospital, and year of delivery. ** Adjusted for maternal age, type of insurance, working status, mode of delivery, preterm birth, prenatal care, multiple birth, maternal comorbidities, type of hospital, and year of delivery. ^†^ Adjusted for maternal age, type of insurance, residential area, working status, preterm birth, prenatal care, multiple birth, maternal comorbidities, type of hospital, and year of delivery. ^‡^ Adjusted for maternal age, type of insurance, residential area, working status, mode of delivery, preterm birth, prenatal care, multiple birth, type of hospital, and year of delivery.
